# Synergistic Effect of Disease Severity, Anxiety Symptoms and Elderly Age on the Quality of Life of Outpatients with Heart Failure

**DOI:** 10.5935/abc.20190174

**Published:** 2020-01

**Authors:** José Henrique Cunha Figueiredo, Gláucia Maria Moraes de Oliveira, Basílio Bragança Pereira, Ana Elisa Bastos Figueiredo, Emília Matos Nascimento, Marcelo Iorio Garcia, Sergio Salles Xavier

**Affiliations:** 1 Universidade Federal do Rio de Janeiro - Cardiologia, Rio de Janeiro, RJ - Brazil; 2 Fundação Oswaldo Cruz, Rio de Janeiro, RJ - Brazil; 3 Centro Universitário Estadual da Zona Oeste, Rio de Janeiro, RJ - Brazil

**Keywords:** Heart Failure, Anxiety/diagnosis, Hospitalization, Quality of Life, Age, Systolic Volume

## Abstract

**Background:**

Heart failure (HF) is a multifactorial syndrome with repercussions on quality of life (QoL).

**Objectives:**

To investigate the main interacting factors responsible to worsen quality of life of outpatients with HF.

**Methods:**

Cross-sectional observational study with 99 patients of both genders, attending a HF outpatient clinic at a university hospital, all with a reduced ejection fraction (<40%) by echocardiography. They were evaluated using sociodemographic and clinical questionnaires, the Minnesota Living with Heart Failure (MLwHF), and the Hospital Anxiety and Depression scale (HADS). QoL was the outcome variable. Two multivariate models were used: the parametric beta regression analysis, and the non-parametric regression tree, considering p < 0.05 and 0.05 < p < 0.10 for statistical and clinical significance, respectively.

**Results:**

Beta regression showed that depression and anxiety symptoms worsened the QoL of HF patients, as well as male sex, age younger than 60 years old, lower education level, lower monthly family income, recurrent hospitalizations and comorbidities such as ischemic heart diseases and arterial hypertension. The regression tree confirmed that NYHA functional class III and IV worsen all dimensions of MLwHF by interacting with anxiety symptoms, which influenced directly or indirectly the presence of poorer total score and emotional dimension of MLwHF. Previous hospitalization in the emotional dimension and age younger than 60 years in general dimension were associated with anxiety and NYHA functional class, also worsening the QoL of HF patients.

**Conclusion:**

HF with reduced ejection fraction was associated with poorer MLwHF. Anxiety symptoms, previous hospitalization and younger age were also associated with worsened MLwHF. Knowledge of these risk factors can therefore guide assessment and treatment of HF patients.

## Introduction

Heart failure (HF) is the leading cause of heart disease morbidity and mortality and is more common among people aged 60 or older.^1^ HF deeply affects the health of an individual, and has physical, psychological and social consequences. HF is a syndrome that severely impairs quality of life (QoL), predisposing patients to recurrent hospitalizations,^2^ and high morbidity and mortality rates, as observed in the Framingham’s study.^3^

In a study^4^ of 204 HF outpatients, the authors found that 46% of the outpatients had depressive and anxiety symptoms at baseline. After a five-year follow-up, even after controlling for disease severity and other risk factors, depressive symptoms were still associated with outcomes such as hospitalization and death.^5^ When analyzing patients who had difficulty in taking medication, the authors pointed out that these patients had more severe HF symptoms and worse quality of life, which can be partially explained by the coexistence of depression and psychological distress such as dysphoria and anxiety.^6^

The association between depression, physical symptoms and QoL in HF patients was observed by Bekelman et al.^7^ in a cross-sectional study with 60 outpatients. It was more common to see patients with depression and anxiety after they suffered dyspnea (OR 5.28, p < 0.05), and those who exhibited more symptoms of depression presented more HF symptoms (p < 0.0001) and poorer quality of life. Another study with patients who had been diagnosed with HF concluded that depression was associated with a worse health status at the baseline, and was a strong predictor of hospitalization, and worse HF symptoms, functional status and QoL.^8^

Physical symptoms are affected by depression and anxiety, as reported in a study^9^ that showed that psychological variables could affect QoL as much as physical symptoms of HF. In a multiple regression analysis, physical symptoms, age, employment status and anxiety at baseline were the best QoL predictors after a three-month follow-up. Depression, perceived control of HF, employment status and younger age were predictors of physical symptom status after three months. The great unanswered question is how these variables interact to worsen patients' QoL, and how much they compromise the therapeutic approach in the various degrees of impairment of ventricular function.

Thus, we collected sociodemographic, clinical variables, anxiety and depression symptoms, medications in use, previous hospitalization and left ventricular ejection fraction (LVEF) to investigate which factors are associated and interact to get worse quality of life of outpatients with heart failure.

## Methods

This study was approved by the Ethics Committee of the Hospital Universitário Clementino Fraga Filho under the protocol number 104/2010. The patients signed a consent form to participate in this study, which included an observational, cross-sectional and descriptive series of consecutive cases. Patients allowed to participate had HF with reduced LVEF < 40^10^ and New York Heart Association (NYHA) functional class I to IV and they were aged ≥ 20 years. Patients with HF caused by valvular dysfunction or reversible causes, and patients who were unable to be interviewed due to psychiatric syndromes, cognitive impairment assessed clinically, and hearing loss were excluded. All participants were recruited at the HF outpatient clinic of the Cardiology Department of the Federal University of Rio de Janeiro from March 2011 to September 2012. One hundred and twenty patients were interviewed individually and a sample of 99 patients of both sexes met the criteria for inclusion.

All participants sociodemographic and clinical questionnaires, the Brazilian version of the Minnesota Living with Heart Failure Questionnaire (MLwHF),^11,12^ and the Hospital Anxiety and Depression Scale (HADS).^13,14^

The sociodemographic questionnaire considered age, sex, monthly family income (dollar), education (years) categorized in illiterate, education 1 (<5 yrs), education 2 (6-12 yrs), education 3 (>12 yrs), marital status (married) and family support. The clinical questionnaire considered NYHA functional class, comorbidities such as atrial fibrillation, chronic renal failure, diabetes mellitus and arterial hypertension; current use of drugs such as betablockers, spironolactone, angiotensin-converting-enzyme inhibitor (ACE), angiotensin AT1 receptor blocker (ARB), nitrate, hydralazine; previous hospitalization and LVEF.

The MLwHF is a structured QoL questionnaire for patients with HF and it has been translated and validated for the Brazilian population.^11,12^ The questions are related to how the patient felt during the 30 days before completing the questionnaire. The MLwHF is made up of 21 questions that address the perception of the *physical* (strongly correlated with dyspnea and fatigue), *emotional* (correlated with emotional and social aspects) and *general well-being* (correlated with financial issues, the side effects of medication and lifestyle), and their scores vary from 0 to 40, 0 to 25 and 0 to 40, respectively. A higher score reflected a worse QoL.

The HADS was developed specifically for use in medically ill populations. It is based on mood, depression and anhedonia and excludes physical symptoms such as sleep disturbance, fatigue and body pain, which can be confused with symptoms of other diseases. The HADS is made up of 14 questions, each with four possible answers, and consists in two subscales - anxiety and depression - of seven items each. The responses refer to how the patients felt in the last seven days, and the sum of each subscale varies from 0 to 21. It has been translated and validated in a Brazilian version, using a cutoff of ≥ 8 in samples of medically ill patients.^13,14^ For the analysis, this cutoff was used as an indication of depression and anxiety, which are referred to in this study as “depression and anxiety symptom”. This variable was dichotomized into “possible anxiety” (8 to 11) and “probable anxiety” (12 to 21), and the same for “possible depression” and “probable depression”.

The outcome variables were the MLwHF dimensions, namely, total score, physical, emotional and general welfare. The independent variables were the sociodemographic variables, clinical variables, anxiety symptoms, depression symptoms, current drugs, previous hospitalization and LVEF.

### Statistical analysis

The continuous variables were presented as mean ± standard deviation (variables normally distributed); or median, first quartile and third quartile (non-normal variables). Data normality was tested using the Shapiro-Wilk normality test. The comparison between the NYHA I/II and NYHA III/IV groups was made using unpaired t-Student test, for normal continuous variables, Wilcoxon rank sum test, for non-normal continuous variables, and Exact Binomial Test, for categorical variables.

Total score, physical, and the emotional and general dimensions of the MLwHF were the outcome variables. The association of the variables described above with the outcomes and dimensions of QoL were evaluated using a parametric beta regression model and a non-parametric regression tree.^15^ Beta regression is a new model recently developed by the Brazilian authors Silvia Ferrari and Francisco Cribari, used when the outcome is a continuous variable that varies in the interval (0,1). The regression tree, apart from its predictive power and its easy visual interpretation, is also extremely useful to find possible interactions between predictive variables, including in situations of unexpected interactions, as in our case. Its final nodes result in the boxplot^16,17^ of the outcome variables. The Betareg package^18^ of the R software^19^ was used. Values of p < 0.05 and 0.05 < p < 0.10 were considered statistically significant and clinical significance, respectively.

Data of 99 patients were analyzed; two did not have the LVEF data, and three did not have data of monthly family income. The missing data were imputed considering the MCAR (missing completely at random) as the missing mechanism of these data.^20^ To facilitate the reading of the figures, the MLwHF scales were adjusted to vary from 0 to 100 (0, 1) and the scores were inverted so the highest scores would be equivalent to better QoL.

## Results

Table 1 describes the characteristics of the sample dichotomized according to the NYHA functional class. Lower LVEF and lower MLwHF, in all dimensions, were significantly more common in NYHA III and IV. On the other hand, family support, absence of comorbidities such as diabetes mellitus, atrial fibrillation, chronic renal failure and the non-use of spironolactone, nitrate or furosemide were significantly more frequent in NYHA I and II.

**Table1:** Characteristics of the sample dichotomized by NYHA functional class

		Total	NYHA = I /II	NYHA = III/IV	p *
Number of patients		99 (100%)	59 (59.60%)	40 (40.40%)	0.0699
**Age**					
Average ± SD		61.05 ± 10.88	59.85 ± 10.65	62.83 ± 11.11	0.1829
**Monthly family income**					
Median (1st Qu.; 3rd Qu.)		914.28 (594.61; 1294.61)	892.3 (594.61; 1564.10)	923.07 (602.56; 1102.56)	0.3978
**Ejection Fraction**					
Average ± SD		35.58 ± 9.18	37.27 ± 8.7	33.1 ± 9.4	0.0258*
**MLwHF Total score**					
Median (1st Qu.; 3rd Qu.)		27 (10.5; 47.0)	17 (5; 32.5)	45 (31.5; 55.0)	< 0.0001***
**MLwHF Physical**					
Median (1st Qu.; 3rd Qu.)		14 (3; 21)	6 (2; 17.5)	20 (15; 25)	< 0.0001***
**MLwHF Emotional**					
Median (1st Qu.; 3rd Qu.)		6 (2; 13)	3 (0.5; 10.5)	10.5 (5.75; 15.25)	0.0001***
**MLwHF General**					
Median (1st Qu.; 3rd Qu.)		7 (3.0; 14.5)	4 (1; 8)	11 (8; 19)	< 0.0001***
Male		61(100%)	38 (62.30%)	23 (37.70%)	0.0722
**Schooling**					
Illiterate		6 (100%)	3 (50.00%)	3 (50.00%)	1.0000
Education (< 5 years)		37 (100%)	24 (64.86%)	13 (35.14%)	0.0989
Education (6-12 years)		52 (100%)	31 (59.62%)	21 (40.38%)	0.2116
Education (> 12 years)		4 (100%)	1 (25.00%)	3 (75.00%)	0.6250
**Married**		64 (100%)	39 (60.94%)	25 (39.06%)	0.1034
**Family support**		82(100%)	51 (62.20%)	31 (37.80%)	0.0352*
**Employed**		19 (100%)	12 (63.16%)	7 (36.84%)	0.3593
**Ischemic Etiology**		39(100%)	21 (53.85%)	18 (46.15%)	0.7493
**Absence of atrial fibrillation**		82(100%)	51 (62.20%)	31 (37.80%)	0.0352*
**Absence of chronic renal failure**		84(100%)	52 (61.90%)	32 (38.10%)	0.0375*
**Absence of diabetes mellitus**		57(100%)	38 (66.67%)	19 (33.33%)	0.0163*
**Arterial hypertension**		69(100%)	39 (56.52%)	30 (43.48%)	0.3356
**Absence of previous hospitalization**		63(100%)	44 (69.84%)	19 (30.16%)	0.0022
**Probable anxiety**		15(100%)	10 (66.67%)	5 (33.33%)	0.3018
**Possible anxiety**		35(100%)	19 (54.29%)	16 (45.71%)	0.7359
**Probable depression**		11(100%)	7 (63.64%)	4 (36.36%)	0.5488
**Possible depression**		27(100%)	15 (55.56%)	12 (44.44%)	0.7011
**Current use of betablockers**		96 (100%)	57 (59.38%)	39 (40.62%)	0.0822
**Absence of spironolactone**		38(100%)	28 (73.68%)	10 (26.32%)	0.0051**
**Current use of ACE inhibitor/ARB**		94(100%)	57 (60.64%)	37 (39.36%)	0.0495*
**Absence Nitrate/Hydralazine**		63(100%)	43 (68.25%)	20 (31.75%)	0.0052**
**Absence Furosemide**		25(100%)	18 (72.00%)	7 (28.00%)	0.0433*
**Optimized treatment**		87(100%)	50 (57.47%)	37 (42.53%)	0.1980

*** p < 0.001;

** p < 0.01;

* p < 0.05.

* P-values were calculated using unpaired t-Student test (for normal continuous variables); Wilcoxon rank sum test (for non-normal continuous variables); and Exact Binomial Test (for categorical variables).

MLwHF: Minnesota Living with Heart Failure; NYHA: New York Heart Association; ACE: Angiotensin-converting enzyme inhibitor; ARB: Angiotensine1 receptor blocker; SD: Standard deviation.

**Figure 1:**
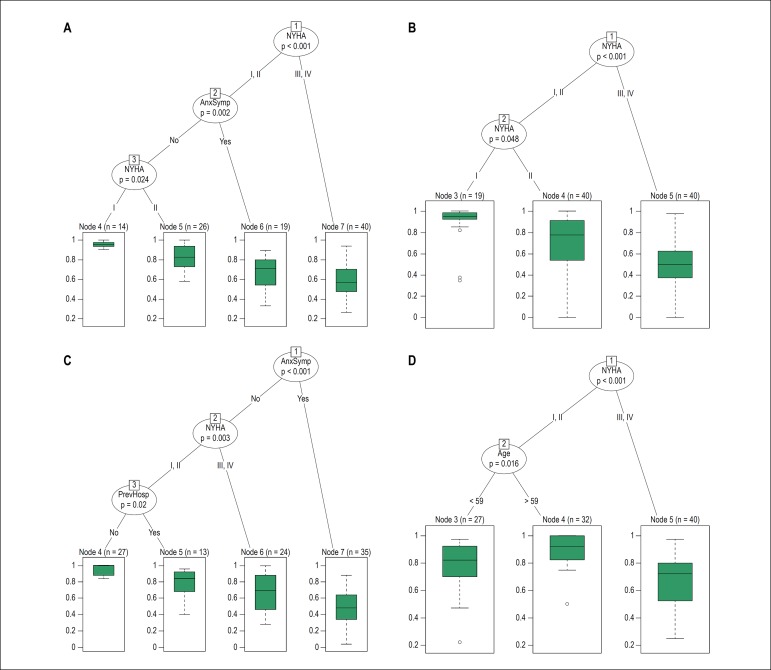
Regression tree (A- Total Score, B- Physical Dimension, C- Emotional Dimension, D-General Dimension) illustrating that advanced NYHA (New York Heart Association) functional class worsened all dimensions of MLwHF (Minnesota Living with Heart Failure). Anxiety symptoms influenced directly or indirectly the presence of poorer total score and emotional well-being dimension of MLwHF. The same was observed for previous hospitalization in the emotional well-being dimension, demonstrating an interaction with functional class NYHA I and II. In the general well-being dimension, the NYHA functional classes I and II were associated with poorer MLwHF in patients younger than 60 years old.

Results of the beta regression analysis are shown in Table 2. It is noteworthy that the predictor variable NYHA functional class has a statistically significant association with all outcome variables, showing that patient’s QoL is affected in all its dimensions by physical symptoms of HF. Anxiety symptoms were also associated with all outcomes except the general well-being dimension. Depression symptoms were associated with the emotional well-being dimension, showing that psychological symptoms affected patients’ QoL in this sample. Previous hospitalization worsened the QoL of outpatients with HF regarding emotional and physical aspects. The current use of medications as betablockers, ACE and furosemide were associated with poor MLwHF in different dimensions. The general well-being dimension of MLwHF decreased with lower monthly family income of the patients. It is worth mentioning that all beta regression models showed a coefficient of determination R2 of about 50% (Figure 1: A-50, B-48, C-56, D-44), which means that the prognostic variables explain 50% of the variation of the outcome or dependent variables.

**Table2:** Beta regression analysis MLwHF and predictor variables

MLwHF QoL Dimensions	Predictor Variables	Estimate (CI 95%)	p
Total score	Education (< 5 years)	0.734 (0.101; 1.366)	0.023 *
	Education (6-12 years)	0.589 (-0.033; 1.211)	0.063
	Education (> 12 years)	0.755 (-0.210; 1.720)	0.125
	Monthly family income	0.000 (0.000; 0.001)	0.019 *
	NYHA II	-0.695 (-1.162; -0.229)	0.003 **
	NYHA III	-1.416 (-1.904; -0.928)	< 0.001 ***
	NYHA IV	-1.404 (-2.066; -0.742)	< 0.001 ***
	Arterial Hypertension	0.585 (0.239; 0.931)	0.001 ***
	Previous hospitalization	-0.553 (-0.898; -0.207)	0.002 **
	Anxiety symptoms	-0.593 (-0.997; -0.190)	0.004 **
	Depression symptoms	-0.402 (-0.828; 0.025)	0.065
	Current Use of Betablocker	0.908 (0.064; 1.752)	0.035 *
Physical	Education (< 5 years)	1.078 (0.223; 1.934)	0.013 *
	Education (6-12 years)	1.077 (0.233; 1.921)	0.012 *
	Education (> 12 years)	1.369 (0.124; 2.614)	0.031 *
	NYHA II	-1.087 (-1.658; -0.516)	< 0.001***
	NYHA III	-1.789 (-2.411; -1.167)	< 0.001***
	NYHA IV	-2.439 (-3.326; -1.552)	< 0.001***
	Arterial Hypertension	0.755 (0.307; 1.203)	0.001 ***
	Previous hospitalization	-0.867 (-1.345; -0.389)	< 0.001***
	Anxiety symptoms	-0.967 (-1.404; -0.529)	< 0.001***
	Current Use of Betablocker	2.018 (0.847; 3.190)	0.001 ***
	Current Use of Furosemide	0.520(0.031; 1.010)	0.037 *
Emotional	Male	0.538 (0.145; 0.932)	0.007 **
	Education (< 5 years)	0.725 (-0.088; 1.539)	0.080
	Education (6-12 years)	0.747 (-0.042; 1.535)	0.063
	Education (> 12 years)	1.135 (-0.099; 2.369)	0.071
	Ischemic etiology	0.561 (0.132; 0.990)	0.010 **
	NYHA II	-0.229 (-0.78; 0.322)	0.416
	NYHA III	-1.234 (-1.818; -0.65)	< 0.001 ***
	NYHA IV	-0.727 (-1.537; 0.083)	0.079
	Previous hospitalization	-0.606 (-1.059; -0.152)	0.009 **
	Anxiety symptoms	-1.104 (-1.614; -0.595)	< 0.001***
	Depression symptoms	-0.879 (-1.420; -0.338)	0.001 ***
	Current Use of ACE	-1.424 (-2.312; -0.536)	0.002 **
General	Male	-0.342 (-0.708; 0.025)	0.068
	Age	0.030(0.013; 0.047)	< 0.001***
	Monthly family income	0.001 (0.000; 0.001)	0.001 ***
	NYHA II	-0.717 (-1.249; -0.184)	0.008 **
	NYHA III	-1.717 (-2.259; -1.176)	< 0.001***
	NYHA IV	-1.895 (-2.627; -1.162)	< 0.001***
	Current Use of Betablocker	1.280(0.323; 2.237)	0.009 **

*** p < 0.001;

** p < 0.01;

* p < 0.05.

MLwHF: Minnesota Living with Heart Failure; NYHA: New York Heart Association; ACE: angiotensin-converting enzyme inhibitor.

The regression tree, the nonparametric model, also evaluated the association of the set of predictor variables with each outcome variable (Figure 1). The results of this model are consonant with the beta model seen in Table 2 and give further information about interactions between the independent variables. Figure 1 illustrates the negative effects of a worse NYHA functional class in all MLwHF dimensions. Anxiety symptoms contributed directly or indirectly to lower total score and poorer emotional well-being in MLwHF. The same was observed for previous hospitalization variable in the emotional well-being dimension, indicating an interaction with NYHA functional classes I and II. In the general well-being dimension, the NYHA classes I and II were associated with poorer MLwHF if patients were younger than 60 years old. Due to inversion of the score system, the interpretation of the regression trees considers that higher values, for each outcome variable, correspond to a better QoL. In other words, we could exemplify that the best quality of life was observed in: NYHA functional class I patients without anxiety symptoms (Figure 1A: total score, 14 patients, 14.1%), NYHA functional class I patients (Figure 1B: physical well-being dimension, 19 patients, 19.2%), NYHA I or II patients with no anxiety symptoms or previous hospitalization (Figure 1C: emotional well-being dimension, 27, 27.3%), and NYHA I or II patients older than 59 years old (Figure 1D: general well-being dimension, 32 patients, 32.3%). We have created a pseudo-coefficient of determination R2 for the regression tree resulting in coefficients around 30% and 40% (Figure 1 A 42% , B 29%, C 44%, D 29%).

## Discussion

The originality of the study consists of the research methods applied to the research question. The regression tree is a nonparametric regression, useful for prediction, and to obtain data not only from the relevant variables, but also from the relevant interactions between these variables. In the usual regression, we can obtain the relevant variables, but we must define what interactions we consider relevant. In the tree of regression, the Data Mining (Machine Learning) algorithm verifies, from the existing data, which variables and which interactions are the most important, providing a better understanding of the complex relationships observed in clinical practice.

This study found an association of ventricular function (represented by NYHA functional class), anxiety symptoms, male sex, age younger than 60 years old, lower education level, lower monthly family income, recurrent hospitalization and comorbidities (such as arterial hypertension and ischemic heart diseases), current use of medications (such as betablockers, ACE and furosemide) with poorer QoL of HF outpatients.

One study^21^ explored the perceptions of QoL in HF patients to assess limitations in their daily lives caused by symptoms, happiness, and relationships, and pointed out that QoL was affected not only by negative physical, psychological, social, and economic status, but also by positive physical, psychological and social status, and behavior, although it was not modified by education combined with self-management intervention. The present study revealed the association of the predictor variable NYHA functional class III-IV with all outcome variables, and of anxiety and depression symptoms with two of the three outcome variables in the beta model. Also, our study shows that even a lower NYHA functional class (I-II), associated with anxiety symptoms and previous hospitalization, may worsen the QoL in the total score and emotional well-being dimension.

HF may be the most devastating chronic disease, as it affects people’s QoL in several dimensions. In fact, other authors have provided evidences that improvements in self-management skills may enhance outcomes of HF patients.^22^

The awareness of one’s own diagnosis has a profound impact on the patients. Mulligan et al.^8^ showed that, addressing patients’ mood and beliefs about their illness and its treatment, improved physical and emotional dimensions of MLwHF and promoted an improvement by 55% and 43% in anxiety and depression symptoms, respectively, assessed by HADS, in the early period after the diagnosis of HF. Reduced anxiety symptoms were associated with reduced perception of the severity of HF and of control due to the treatment of the disease. Reduced depression symptoms was attributed to the improvement of NYHA functional class, reduction in symptoms and perceived severity of HF, and increased confidence in the treatment. The present study confirms these findings that depression and anxiety symptoms contribute to a worsening of QoL related to physical symptoms of HF. It also confirms the fact that when HF patients are less symptomatic and have no anxiety symptoms and recurrent hospitalizations, patient’s QoL is better.

In another study,^23^ less social support and greater depressive symptoms independently predicted poorer QoL. Also, patient’s perspective on family functioning and autonomy support, along with family knowledge about HF, influenced psychological outcomes of depressive symptoms and emotional QoL of patients with HF.^24^ These studies corroborate our results, since married people with family support had positive perception of HF control, and anxiety and depression symptoms directly and negatively affected HF-related physical symptoms, which, in turn, interfered with QoL of these patients.

A meta-analysis concluded that somatic/affective depression symptoms were more strongly and consistently associated with mortality and cardiovascular events in patients with heart disease compared with cognitive/affective symptoms.^25^ Another study^26^ with 55 congestive HF patients also showed that somatic/affective depressive symptoms, but not cognitive depression symptoms and anxiety symptoms, were associated with poor health-related QoL and behavioral functional capacity, independent of age, clinical functional status and comorbidities.^26^ Our study disagreed with these results because NYHA functional class III and IV worsened all dimensions of MLwHF, and anxiety symptoms, combined with age under 60 years, influenced directly or indirectly the presence of poorer score.

A willing attitude toward following a low sodium diet, and an increased social support were significantly associated with higher levels of perceived control and better QoL.^27^ A recent study^28^ pointed out that depressive symptoms exerted a negative effect on medication adherence related to the complexity of medication regimen commonly prescribed to HF patients. In the present study, we also observed that the current use of medications as betablockers, ACE and furosemide were associated with poor MLwHF in different dimensions, suggesting that it could be a barrier to reach medication adherence by HF patients.

How and by what mechanisms such associations are given are not clear. Probably physiological and behavioral factors including endothelial dysfunction, platelet abnormalities, inflammation, autonomic nervous system dysfunction, and reduced engagement in health-promoting activities, may link depression and anxiety with adverse cardiac outcomes and poorer QoL in HF.^29,30^

This study has some limitations. This was a single-center study, although it can encourage future studies in other specialized centers. Also, its cross-sectional design made it difficult to assess possible changes in patients’ QoL and what factors contributed to such changes and to make speculations that would only be possible with a longitudinal design. Thus, generalizations must be made with caution.

## Conclusion

Based on these findings, it is possible to conclude that a reduced LVEF is associated with many factors that compromise the QoL of HF outpatients, even in clinically less severe cases, like in NYHA functional class I or II . These findings may aid in the full approach of patients with HF, suggesting the diagnosis and treatment of anxiety symptoms, especially those with multiple hospitalizations and younger than 60 years.

## References

[1] Roger VL (2013). Epidemiology of Heart Failure. Circ Res.

[2] Ramos S, Prata J, Gonçalves FR, Coelho R (2016). Depression predicts mortality and hospitalization in heart failure: A six-years follow-up study. J Affect Disord.

[3] Mahmood SS, Wang TJ (2013). The epidemiology of congestive heart failure: the Framingham Heart Study perspective. Glob Heart.

[4] Sherwood A, Blumenthal JA, Trivedi R, Johnson KS, O'Connor CM, Adams KF Jr (2007). Relationship of depression to death or hospitalization in patients with heart failure. Arch Intern Med.

[5] Sherwood A, Blumenthal JA, Hinderliter AL, Koch GG, Adams KF Jr, Dupree CS (2011). Worsening depressive symptoms are associated with adverse clinical outcomes in patients with heart failure. J Am Coll Cardiol.

[6] Hallas CN, Wray J, Andreou P, Banner NR (2011). Depression and perceptions about heart failure predict quality of life in patients with advanced heart failure. Heart Lung.

[7] Bekelman DB, Havranek EP, Becker DM, Kutner JS, Peterson PN, Wittstein IS (2007). Symptoms, depression, and quality of life in patients with heart failure. J Card Fail.

[8] Mulligan K, Mehta PA, Fteropoulli T, Dubrey SW, McIntyre HF, McDonagh TA (2012). Newly diagnosed heart failure: Change in quality of life, mood, and illness beliefs in the first 6 months after diagnosis. Br J Health Psychol.

[9] Heo S, Doering LV, Widener J, Moser DK (2015). Predictors and effect of physical symptom status on health-related quality of life in patients with heart failure. Eur J Cardiovasc Nurs.

[10] Ponikowski P, Voors AA, Anker SD, Bueno H, Cleland JG, Coats AJ (2016). ESC Guidelines for the diagnosis and treatment of acute and chronic heart failure: The Task Force for the diagnosis and treatment of acute and chronic heart failure of the European Society of Cardiology (ESC) Developed with the special contribution of the Heart Failure Association (HFA) of the ESC. Eur J Heart Fail.

[11] Rector TS, Kubo SH, Cohn JN (1987). Patients' self-assessment of their congestive heart failure: content, reliability and validity of a new measure, the Minnesota Living with Heart Failure Questionnaire. Heart Fail.

[12] Carvalho VO, Guimarães GV, Carrara D, Bacal F, Bocchi EA (2011). Validação da versão em português do Minnesota Living with Heart Failure Questionnaire. Arq Bras Cardiol.

[13] Zigmond AS, Snaith RP (1983). The Hospital Anxiety and Depression Scale. Acta Psychiatr Scand.

[14] Botega NJ, Bio MR, Zomignani A, Garcia Jr C, Pereira WA (1995). Transtornos do humor em enfermaria de clínica médica e validação de escala de medida (HAD) de ansiedade e depressão. Rev Saude Publica.

[15] Ferrari SL, Cribari-Neto F (2004). Beta-Regression for modelling rates and proportions. J Appl Statistics.

[16] Hothorn T, Hornik K, Zeileis A (2006). Unbiased recursive partitioning: A conditional inference framework. J Comput Graphical Statistics.

[17] Efron B, Hastie T (2016). Computer Age Statistical Inference: Algorithms, Evidence, and Data Science.

[18] Cribari-Neto F, Zeileis A (2010). Beta Regressione in R. J Statistical Software;.

[19] R Core Team (2018). R: A language and environment for statistical computing.

[20] van Buuren S, Groothuis-Oudshoorn K (2011). MICE: Multivariate Imputation by Chained Equations in R. J Statistical Software.

[21] Grady KL, de Leon CFM, Kozak AT, Cursio JF, Richardson J, Avery E (2014). Does Self-management Counseling in Patients with Heart Failure Improve Quality of Life? Findings from the Heart Failure Adherence and Retention Trial (HART). Qual Life Res.

[22] Musekamp G, Schuler M, Seekatz B, Bengel J, Faller H, Meng K (2017). Does improvement in self-management skills predict improvement in quality of life and depressive symptoms? A prospective study in patients with heartfailure up to one year after self-management education. BMC Cardiovasc Disord.

[23] Chung ML, Mosor DK, Terry A, Lennie TA, Susan K, Frazier SK (2013). Perceived social support predicted quality of life in patients withheart failure, but the effect is mediated by depressive symptoms. Qual Life Res.

[24] Stamp KD, Dunbar SB, Clark PC, Reilly CM, Gary RA, Higgins M (2014). Family Context Influences Psychological Outcomes of Depressive Symptoms and Emotional Quality of Life in Patients with Heart Failure. J Cardiovasc Nurs.

[25] Azevedo RM, Roestn AM, Hoen PW, Jonge P (2014). Cognitive/affective and somatic/ affective symptoms of depression in patients with heart disease and their association with cardiovascular prognosis: a meta-analysis. Psychol Med.

[26] Patron E, Benvenuti SM, Lopriore V, Aratari J, Palomba D (2017). Somatic-Affective, But Not Cognitive, Depressive Symptoms are Associated with Reduced Health-Related Quality of Life in Patients with Congestive Heart Failure Heart failure and health related quality of life. Psychosomatics.

[27] Heo S, Lennie TA, Pressler SJ, Dunbar SB, Chung ML, Moser DK (2015). Factors Associated with Perceived Control and the Relationship to Quality of Life in Patients with Heart Failure. Eur J Cardiovasc Nurs.

[28] Goldstein CM, Gathright EC, Gunstad J, Dolansky MA, Redle JD, Josephson R (2017). Depressive symptoms moderate the relationship between medication regimen complexity and objectively measured medication adherence in adults with heart failure. J Behav Med.

[29] Bradley SM, Rumsfeld JS (2015). Depression and cardiovascular disease. Trends Cardiovasc Med.

[30] Streng KW, Nauta JF, Hillege HL, Anker SD, Cleland JG, Dickstein K (2018). Non-cardiac comorbidities in heart failure with reduced, mid-range and preserved ejection fraction. Int J Cardiol.

